# Electron transfer dynamics and electrocatalytic oxygen evolution activities of the Co_3_O_4_ nanoparticles attached to indium tin oxide by self-assembled monolayers

**DOI:** 10.3389/fchem.2022.919192

**Published:** 2022-08-24

**Authors:** Xuan Liu, Qianhong Tian, Yvpei Li, Zixiang Zhou, Jinlian Wang, Shuling Liu, Chao Wang

**Affiliations:** Shaanxi Collaborative Innovation Center of Industrial Auxiliary Chemistry and Technology, Department of Chemistry and Chemical Engineering, Key Laboratory of Auxiliary Chemistry and Technology for Chemical Industry, Ministry of Education, Shaanxi University of Science and Technology, Xi’an, Shaanxi, China

**Keywords:** dicarboxylic acid, spinel cobalt oxide nanoparticles, oxygen evolution reaction, electron transfer kinetics, indium tin oxide

## Abstract

The Co_3_O_4_ nanoparticle-modified indium tin oxide-coated glass slide (ITO) electrodes are successfully prepared using dicarboxylic acid as the self-assembled monolayer through a surface esterification reaction. The ITO-SAM-Co_3_O_4_ (SAM = dicarboxylic acid) are active to electrochemically catalyze oxygen evolution reaction (OER) in acid. The most active assembly, with Co loading at 3.31 × 10^−8^ mol cm^−2^, exhibits 374 mV onset overpotential and 497 mV overpotential to reach 1 mA cm^−2^ OER current in 0.1 M HClO_4_. The electron transfer rate constant (*k*) is acquired using Laviron’s approach, and the results show that *k* is not affected by the carbon chain lengths of the SAM (up to 18 -CH_2_ groups) and that an increase in the average diameter of Co_3_O_4_ nanoparticles enhances the *k*. In addition, shorter carbon chains and smaller Co_3_O_4_ nanoparticles can increase the turn-over frequency (TOF) of Co sites toward OER. The Co_3_O_4_ nanoparticles tethered to the ITO surface show both a higher number of electrochemically active Co sites and a higher TOF of OER than the Co_3_O_4_ nanoparticles bound to ITO using Nafion.

## Introduction

Electrodes with self-assembled monolayers (SAMs) have broad applications in the fields of sensing, catalysis, and energy storage and conversion. Understanding the electron transfer dynamics between the electrode substrate and the attached monolayer is crucial in designing interfaces for these applications (Kellon et al., 2019; Xie et al., 2019). Electrodes with tethered nanoparticles by the SAM feature low loadings and high atomic utilization of nanoparticles, both of which are desirable in the field of catalysis (Young et al., 2016; Shermukhamedov et al., 2020; Lachmanová et al., 2021). Chazalviel and Allongue (2011) established a theory in describing the relationship between the insulating layer (SAM) thickness and electron transfer dynamics in the substrate-SAM-nanoparticle, where the electron transfer rate is unhindered until a threshold thickness of the SAM is reached. Later, Hill et al., 2015 proposed a model to qualitatively calculate the current of the substrate-SAM-nanoparticle assembly by considering the electron tunneling, reaction kinetics, and mass transport (Hill et al., 2015). Evidences supporting these theories using gold nanoparticles as the electron mediator are extensively reported (Wang et al., 2010; Barfidokht et al., 2013; Kizling et al., 2018; Liu et al., 2019). However, gold nanoparticles are catalytically inert in most cases, and nanoparticles with catalytic activities are desirable to be assembled onto the electrode surface in catalysis.

The main bottle-neck of hydrogen production by electrochemical water splitting is the anodic oxygen evolution reaction (OER, 2 H_2_O → 4 H^+^ + O_2_ + 4 e^−^, *E* ^o^ = 1.23 V). The four coupled electron and proton transfer processes cause the sluggish kinetics of the OER, and active electrocatalysts and interfaces are required to reduce the large overpotential of the OER (Walter et al., 2010). The nanoparticle-attached electrodes have been constructed via SAM for OER. For example, iridium oxide nanoparticles (IrO_x_) and ruthenium oxide nanoparticles (RuO_x_) have been attached to the indium tin oxide-coated glass slide (ITO) surface *via* esterification reaction using polycarboxylic acids and pyrophosphoric acid as the linker, and the resulting electrodes are applied to catalyze the OER in acid (Gambardella et al., 2012; Tian et al., 2021). Consistent with the theory established by Chazalviel and Allongue (2011) the apparent electron transfer rates of the IrO_x_-modified electrodes are unhindered using the linkers with short carbon-chains, and the electron tunneling rate does not limit the OER rate (Gambardella et al., 2012; Tian et al., 2021). Since iridium is a precious metal, interfaces with nonprecious metal nanoparticles are highly desirable to catalyze the OER. Spinel-type cobalt oxide nanoparticles (Co_3_O_4_) have been adopted to catalyze the OER in acid and are a promising candidate in replacing in part the precious metal catalysts (Lai et al., 2021; Natarajan et al., 2021). Co_3_O_4_ is usually electrodeposited on the substrate surface or is bonded to the substrate surface using Nafion (Liu et al., 2013). These methods inevitably lead to low percentage of utilization of electrochemically active Co atoms as the active sites, as some nanoparticles are not in direct contact with the electrolyte. The substrate-SAM-Co_3_O_4_ assembly offers a promising route in fine-tuning the surface structure of the catalytic active surface, with a maximum percentage of Co exposed. Therefore, we report the construction of the ITO-SAM-Co_3_O_4_ (SAM = dicarboxylic acid) assembly, and the constructed interface is active toward the OER in acid. The influences of SAM chain lengths and the average diameters of the Co_3_O_4_ on the electron transfer kinetics and on the electrocatalytic OER activities are investigated. Also, comparisons to the electrode with Nafion-bound Co_3_O_4_ are made, and the Co_3_O_4_ tethered by SAM show significantly enhanced number of electrochemically active Co sites and increased OER activity per active site.

## Experimental section

### Preparation of Co_3_O_4_

Chemicals used are listed in the Supplementary Information. The surfactant-free Co_3_O_4_ were synthesized based on the literature report (Dong et al., 2007). The procedure to prepare Co_3_O_4_ with an average diameter of 3.5 nm is as follows. The 0.5 g cobalt (II) acetate tetrahydrate (Co(ac)_2_ 4 H_2_O) was dissolved in 25 ml ethanol, and 2.5 ml 25% NH_3_·H_2_O was added under vigorous stirring. The solution was stirred for 10 min in air and was transferred into a Teflon-lined stainless-steel. The autoclave was kept at 150°C for 3 h. The colloidal solution was centrifuged at 10,000 rpm for 15 min to acquire the Co_3_O_4_ precipitates. The precipitates were washed twice with distilled water and dried in the oven at 60°C for 4 h. The Co_3_O_4_ with various sizes were synthesized following the same procedure, but different amounts of the reactants (Co(ac)_2_ 4 H_2_O, C_2_H_5_OH, H_2_O, and NH_3_·H_2_O) were added according to Supplementary Table S1. The XRD and TEM images of the as-synthesized Co_3_O_4_ with various sizes are shown in the Supplementary Information (Supplementary Figures S1, S3).

### Preparation of the ITO-SAM-Co_3_O_4_

The ITO was cleaned with water and ethanol under sonication and was dried. Then, the ITO was immersed in 5 ml of acetone containing dicarboxylic acid (glutaric acid, 1,8-octanedioic acid, 1,16-hexadecanedioic acid, or 1,20-eicosanedioic acid) for 3 h to allow the adsorption of the acid onto the ITO surface. The ITO-SAM (ITO-Glu, ITO-Oct, ITO-Hex, or ITO-Eic) was washed with acetone and dried in air. The acid-adsorbed ITO was immersed in the 5 ml colloidal solution (pH adjusted to 2.0 using HCl) containing 3.5 nm Co_3_O_4_ (4.6 mg ml^−1^) for 4 h. After being taken out from the solution, the ITO-SAM-Co_3_O_4_ was washed with 0.1 M HClO_4_. Electrodes prepared using glutaric acid, 1,8-octanedioic acid, 1,16-hexadecanedioic acid, and 1,20-eicosanedioic acid are labeled as ITO-Glu-Co_3_O_4_, ITO-Oct-Co_3_O_4_, ITO-Hex-Co_3_O_4_, and ITO-Eic-Co_3_O_4_, respectively. The concentrations of the carboxylic acids in acetone are listed in Supplementary Table S2 in the Supplementary Information. Surface loadings of Co are checked by ICP-AES and are in the range of 2.0–5.5 × 10^−8^ mol cm^−2^.

To prepare ITO-Oct-Co_3_O_4_ with different sizes of Co_3_O_4_ attached, the ITO-Oct was immersed in a 5 ml colloidal solution (pH 2.0) containing Co_3_O_4_ with different sizes for 4 h. Electrodes prepared using Co_3_O_4_ with average diameters of 11 and 19 nm are labeled as ITO-Oct-Co_3_O_4_ (11) and (19), respectively. The ITO-Co_3_O_4_ was prepared by directly immersing a bare ITO into a Co_3_O_4_ colloidal solution for 4 h, and the ITO-Oct-CoCl_2_ was fabricated by immersing the ITO-Oct in a 5 ml 0.0016 M CoCl_2_ aqueous solution.

### Preparation of the Co_3_O_4_/Nafion electrode

The ink was prepared by dispersing 0.0011 g Co_3_O_4_ with 3.5 nm average diameter into 20 ml of absolute ethanol, with 30 μl of 0.5% Nafion added. After sonication for 30 min, 50 μl of the solution was drop-coated on the ITO surface with ∼1 cm^2^ covered. After being dried in air, the Co_3_O_4_/Nafion electrode is used for electrochemical tests. The loading of the Co on the ITO-Oct-Co_3_O_4_ and Co_3_O_4_/Nafion electrodes is the same (3.31 × 10^−8^ mol cm^−2^).

### Electrochemical method

The CHI660E and ParSTAT MC potentiostats are used to carry out the electrochemical tests in the three-electrode system. A saturated calomel electrode (SCE) is used as the reference and a polished graphite rod as the counter. The geometric surface area of the working electrode in the electrolyte is controlled to 1 cm^2^. All potentials reported are relative to the reversible hydrogen electrode. The linear sweep voltammograms (LSVs) are corrected for the solution resistance. Other information about the instrumentation is provided in the Supplementary Information.

## Results and discussion

**SCHEME 1:**
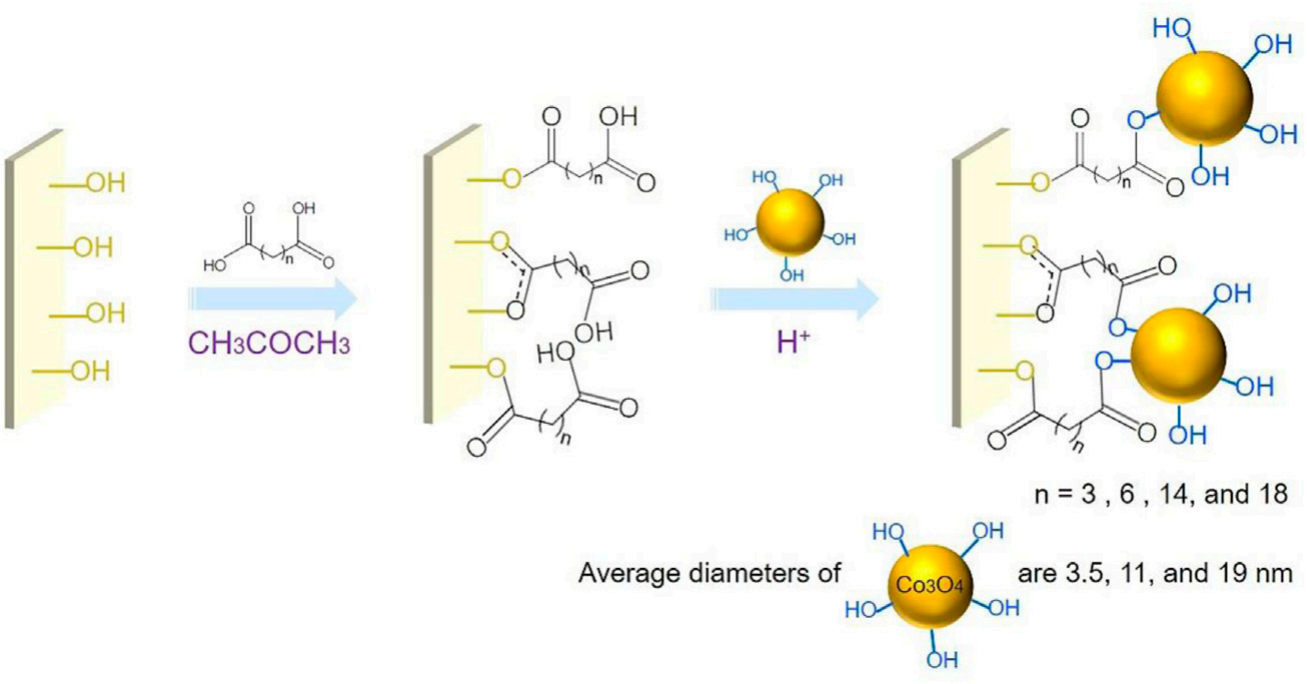
Procedure to prepare ITO-SAM-Co_3_O_4_ (SAM = dicarboxylic acid).

Scheme 1 illustrates the procedure to fabricate the ITO-SAM-Co_3_O_4_ (SAM = dicarboxylic acid). In acetone solution containing the dicarboxylic acid, surface hydroxyl groups of ITO can react with the carboxylic acid groups to form ester bonds. Then, the ITO-SAM (SAM = dicarboxylic acid) is immersed in the acidic solution containing Co_3_O_4_ to allow the esterification between the adsorbed carboxylic acid groups and the hydroxyl groups of Co_3_O_4_. Hydroxyl groups on the Co_3_O_4_ surface have been observed previously (Anantharaj et al., 2019), and the interaction of carboxylic acid groups with the Co_3_O_4_ surface hydroxyl groups has also been reported (Kollhoff et al., 2018). Dicarboxylic acids with different numbers of carbon chains are adopted. The Co_3_O_4_ with various average diameters (3.5, 11, and 19 nm) are synthesized, and the TEM (Supplementary Figure S3) and XRD (Supplementary Figure S1) of the Co_3_O_4_ powders are shown in the Supplementary Information. All XRD patterns show diffraction peaks that correspond to the face-centered cubic phase of Co_3_O_4_ (JCPDS 09-0418) (Dong et al., 2007), and Co_3_O_4_ with larger average diameters show increased XRD peak intensities, which suggests their better crystallinities. The pH of the Co_3_O_4_-containing solution (pH = 2.0) is lower than the p*K*a_1_ values of the polycarboxylic acids adopted [glutaric acid 4.34 (Canari and Eyal, 2003), 1,8-octanedioic acid 4.5 (Hullar and Anastasio, 2011), 1,16-hexadecanedioic acid 4.65 (Kanicky and Shah, 2002), and 1,20-eicosanedioic acid 5.5 (Mukerjee and Ostrow, 2010)]; therefore, carboxylic acid groups remain in the -COOH form to allow the esterification reaction to happen.

**FIGURE 1:**
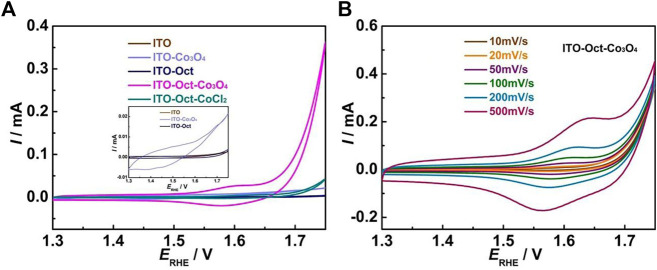
**(A)** CV of the bare ITO, ITO-Oct, ITO-Co_3_O_4_, ITO-Oct-Co_3_O_4_, and ITO-Oct-CoCl_2_ at 50 mV s^−1^ in 0.1 M HClO_4_; **(B)** CV of the ITO-Oct-Co_3_O_4_ at various scan rates (10–500 mV s^−1^) in 0.1 M HClO_4_.

We first used cyclic voltammetry (CV) to probe the surface change during the fabrication of the ITO-Oct-Co_3_O_4_. Figure 1 shows the results. For the ITO-Oct-Co_3_O_4_, a couple of redox peaks are shown at ∼ 1.58 V, which can be assigned to the Co^3+/4+^ redox couple in acid (Xiao et al., 2020; Han et al., 2021; Natarajan et al., 2021). The charge under the anodic Co^3+/4+^ peak is integrated, and the number of electrochemically active Co atoms (*Γ*) is estimated to be 4.6 × 10^−10^ mol cm^−2^ based on the integrated charge, assuming a 1 e^−^ transfer process. The total amount of Co on the ITO-Oct-Co_3_O_4_ is 3.31 × 10^−8^ mol cm^−2^ acquired using ICP-AES. These values indicate that 1.4% of the Co on the electrode is electrochemically active. Starting at 1.65 V, the OER process happens. In comparison, the CV of the bare ITO and ITO-Oct is identical (Figure 1A inset) and lack the characteristic Co^3+/4+^ redox peaks and the OER process. Electrodes prepared by simply immersing the ITO into the Co_3_O_4_ colloidal solution (ITO-Co_3_O_4_) give rise to a higher CV current than bare ITO and show redox features, but the current is still negligible compared to the ITO-Oct-Co_3_O_4_. We also observed a strong dependence of the coverage of the Co_3_O_4_ on the pH of the esterification reaction (Supplementary Figure S4), which implies that the ester bonds formed are the major driving force to anchor the Co_3_O_4_ to the surface, while the ionic interaction, hydrogen bonding, and chelation between the acid and the defected cationic centers contribute insignificantly to the surface bonding as they are less dependent on pH. Immersing the ITO-Oct in CoCl_2_ solution (ITO-Oct-CoCl_2_) leads to formation of Co^2+^ coordinated to the carboxylic acid groups at the surface, and the CV lacks the unique feature for Co^3+/4+^ redox couple but shows a slight increase in the OER current at high potentials. These show that the Co_3_O_4_, rather than Co ionic species, are tethered to the ITO in case of the ITO-Oct-Co_3_O_4_. Figure 1B displays the CV of the ITO-Oct-Co_3_O_4_ at various scan rates in 0.1 M HClO_4_. All the CV show distinct Co^3+/4+^ redox peaks, and by plotting the log *i*_p_ versus log *v* (Supplementary Figure S5), the slope is close to 1, which implies that the redox active species are confined to the electrode surface (Silva et al., 2014).

**FIGURE 2:**
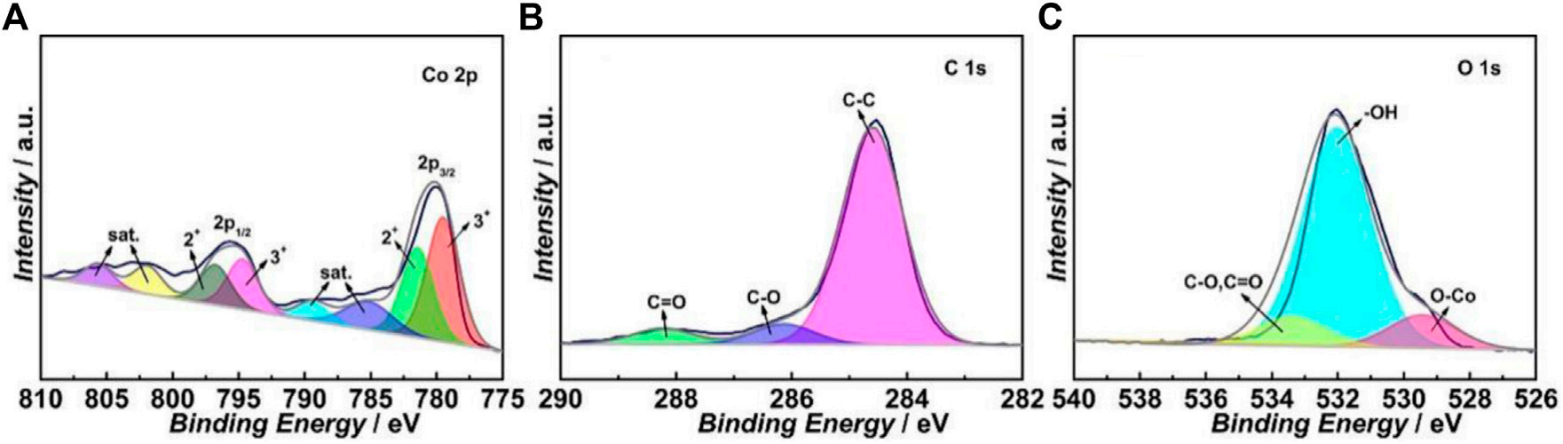
Deconvoluted high-resolution XPS spectra of the **(A)** Co 2p, **(B)** C 1s, and **(C)** O 1s regions of the ITO-Oct-Co_3_O_4_.

Figure 2A shows the deconvoluted XPS spectrum of the Co 2p region of the ITO-Oct-Co_3_O_4_. The Co 2p_3/2_ peak can be deconvoluted into two components at 781.5 and 779.5 eV, which originate from the Co^2+^ and Co^3+^ in the Co_3_O_4_, respectively (Zhang et al., 2018). The deconvoluted Co 2p_1/2_ peak also shows the contribution from the Co^2+^ (796.8 eV) and Co^3+^ (794.7 eV) components. The deconvoluted C 1s spectrum in Figure 2B displays three peaks at 284.6, 286.1, and 288.2 eV, which correspond to the C-C, C-O, and C=O bonds, respectively (Dwivedi et al., 2015). The deconvoluted O 1s peak in Figure 2C shows three components at 529.4, 532.0, and 533.4 eV, which match the Co-O bond in the Co_3_O_4_, surface -OH structure, and the C-O and C=O bonds, respectively (Yang et al., 2009). The existence of C=O and C-O structures in both C 1s and O 1s spectra indicates the formation of ester bonds. The XRD pattern of the ITO-Oct-Co_3_O_4_ shows only crystalline peaks assigned to the ITO substrate (Supplementary Figure S2), suggesting the loading of crystalline Co_3_O_4_ is low. Both the XPS and CV show that the Co_3_O_4_ are successfully tethered to the ITO surface using the dicarboxylic acid as SAM.

**FIGURE 3:**
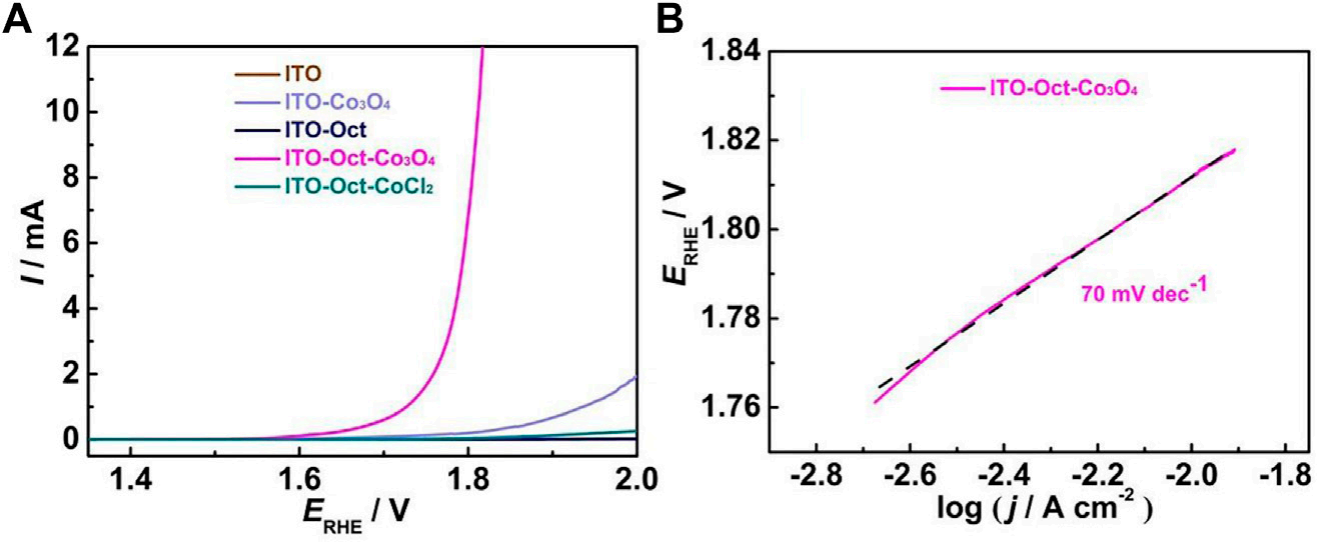
**(A)** LSV of the bare ITO, ITO-Oct, ITO-Co_3_O_4_, ITO-Oct-Co_3_O_4_, and ITO-Oct-CoCl_2_ at 5 mV s^−1^ in 0.1 M HClO_4_; **(B)** Tafel plot of the ITO-Oct-Co_3_O_4_.

Figure 3A compares the OER activity of the ITO-Oct-Co_3_O_4_ with bare ITO, ITO-Oct, ITO-Co_3_O_4_, and ITO-Oct-CoCl_2_ in 0.1 M HClO_4_ at 5 mV s^−1^. Significantly higher OER current is observed at the high-potential region (>1.65 V) for the ITO-Oct-Co_3_O_4_, while the ITO and ITO-Oct exhibit negligible current in the similar region. This indicates that the attached Co_3_O_4_ is responsible for the observed OER activity. The OER onset overpotential of the ITO-Oct-Co_3_O_4_ is 374 mV, and the overpotential to reach 1, 5, and 10 mA cm^−2^ current densities is 497, 562, and 570 mV, respectively. The Tafel plot and the corresponding Tafel slope value of the ITO-Oct-Co_3_O_4_ are displayed in Figure 3B. The Tafel slope value acquired is 70 mV dec^−1^, consistent with the reported Tafel slopes of Co_3_O_4_ in acid (Mondschein et al., 2017; Han et al., 2021). The turn-over frequency (TOF) at 1.72 V is calculated based on Eq. 1

TOF=j/4FΓ (1)

to be 4.06 s^−1^. The key electrochemical parameters of the ITO-Oct-Co_3_O_4_ are summarized in Table 1.

**TABLE 1:** Summary of the key electrochemical parameters of the ITO-SAM-Co_3_O_4_ (SAM = dicarboxylic acid).

	SAM	*Г*_cv_/nmol cm^−2^	*η*_onset_/mV	*η*@1 mA cm^−2^/mV	*η*@5 mA cm^−2^/mV	*Tafel Slope*/mV dec^−1^	*TOF*@1.72 V/s^−1^	*k*/s^−1^
ITO-Glu- Co_3_O_4_	HOOC(CH_2_)_3_COOH	0.39	433 ± 1	514 ± 1	597 ± 4	98	3.87 ± 0.03	1.12 ± 0.07
ITO-Oct-Co_3_O_4_	HOOC(CH_2_)_6_COOH	0.46	374 ± 4	497 ± 2	562 ± 3	70	4.06 ± 0.04	1.11 ± 0.04
ITO-Hex-Co_3_O_4_	HOOC(CH_2_)_14_COOH	0.55	402 ± 1	514 ± 1	590 ± 4	109	2.70 ± 0.01	1.10 ± 0.02
ITO-Eic-Co_3_O_4_	HOOC(CH_2_)_18_COOH	0.32	444 ± 1	529 ± 1	598 ± 6	91	2.87 ± 0.01	1.14 ± 0.08
ITO-Oct-Co_3_O_4_ (11)	HOOC(CH_2_)_6_COOH	0.33	449 ± 2	525 ± 1	590 ± 3	112	3.02 ± 0.07	1.15 ± 0.09
ITO-Oct-Co_3_O_4_ (19)	HOOC(CH_2_)_6_COOH	0.33	451 ± 1	533 ± 1	623 ± 2	84	2.88 ± 0.04	1.35 ± 0.07

**FIGURE 4:**
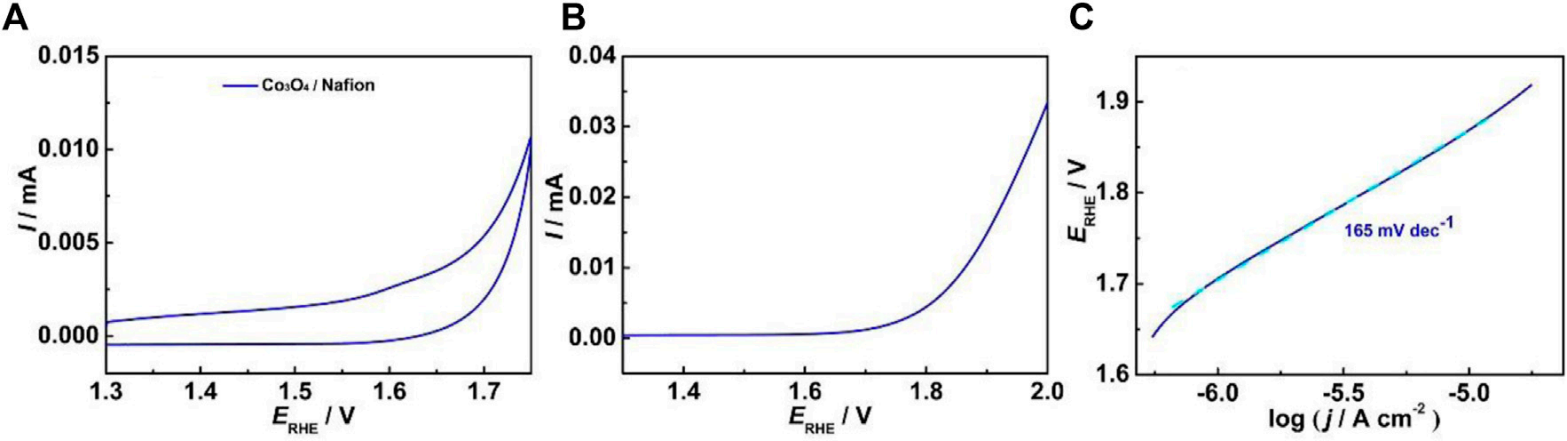
**(A)** CV of the Co_3_O_4_ bound by Nafion on ITO at 50 mV s^−1^ in 0.1 M HClO_4_; **(B)** LSV of the electrode at 5 mV s^−1^ in 0.1 M HClO_4_; **(C)** Tafel plot of the electrode.

We also prepared the Co_3_O_4_ (3.5 nm in average diameter bound by Nafion on ITO, and the amount of Co deposited on the ITO is controlled to 3.31 × 10^−8^ mol cm^−2^, same as the ITO-Oct-Co_3_O_4_. Figure 4A shows the CV of the electrode at 50 mV s^−1^ in 0.1 M HClO_4_. Weak Co^3+/4+^ redox peaks are shown centered at 1.63 V. By integrating the charge under the Co^3+/4+^ anodic peak of the Co_3_O_4_/Nafion electrode, assuming a 1 e^−^ transfer process, the *Γ* is estimated to be 6.66 × 10^−11^ mol cm^−2^. This indicates that the ITO-Oct-Co_3_O_4_ exposes seven times higher amount of the electrochemically active Co atoms than the Co_3_O_4_/Nafion electrode with the same Co_3_O_4_ loading. This is consistent with previous observations using IrO_x_ and Au nanoparticles and can be caused by the inhomogeneous distribution of Nafion bound Co_3_O_4_ at the ITO surface, as aggregation is constantly observed on the Nafion bound nanoparticles (Moghaddam et al., 2015). In addition, different substrate–nanoparticle interactions and parallel interactions can account for the observed differences in the *Γ*. Figure 4B shows the LSV of the Co_3_O_4_/Nafion electrode. The OER process is also observed starting ∼480 mV overpotential, but the OER current at 1.72 V is significantly lower than that of the ITO-Oct-Co_3_O_4_, in part attributed to the lower *Γ*. The TOF at 1.72 V for the Co_3_O_4_/Nafion electrode is calculated to be 0.058 s^−1^, 69 times lower than that of the ITO-Oct-Co_3_O_4_. Figure 4C shows the Tafel plot of the Co_3_O_4_/Nafion electrode. The Tafel slope value of the Co_3_O_4_/Nafion electrode is 165 mV dec^−1^. This suggests sluggish OER kinetics of the Co_3_O_4_/Nafion electrode. Both the lower TOF and the higher Tafel slope value for the Co_3_O_4_/Nafion electrode indicate inferior electrocatalytic activity per active Co site for the electrode with Nafion-bound Co_3_O_4_. As we adopted the same Co_3_O_4_, the surface structure of the active sites is similar. The observed inferior activity per active Co site is probably caused by the hindered electron or proton transport for the OER process. This proposal agrees with the results acquired by Young et al. (2016), where molecular tether facilitates the Au nanoparticle-mediated electron transfer process (Young et al., 2016). Therefore, the dicarboxylic acid-tethered Co_3_O_4_ exposed significantly higher amount of electrochemically active Co atoms than the Nafion bound Co_3_O_4_, and the electrocatalytic OER activities of nanoparticles acquired using the Nafion as the binder can be a severe underestimation.

**FIGURE 5:**
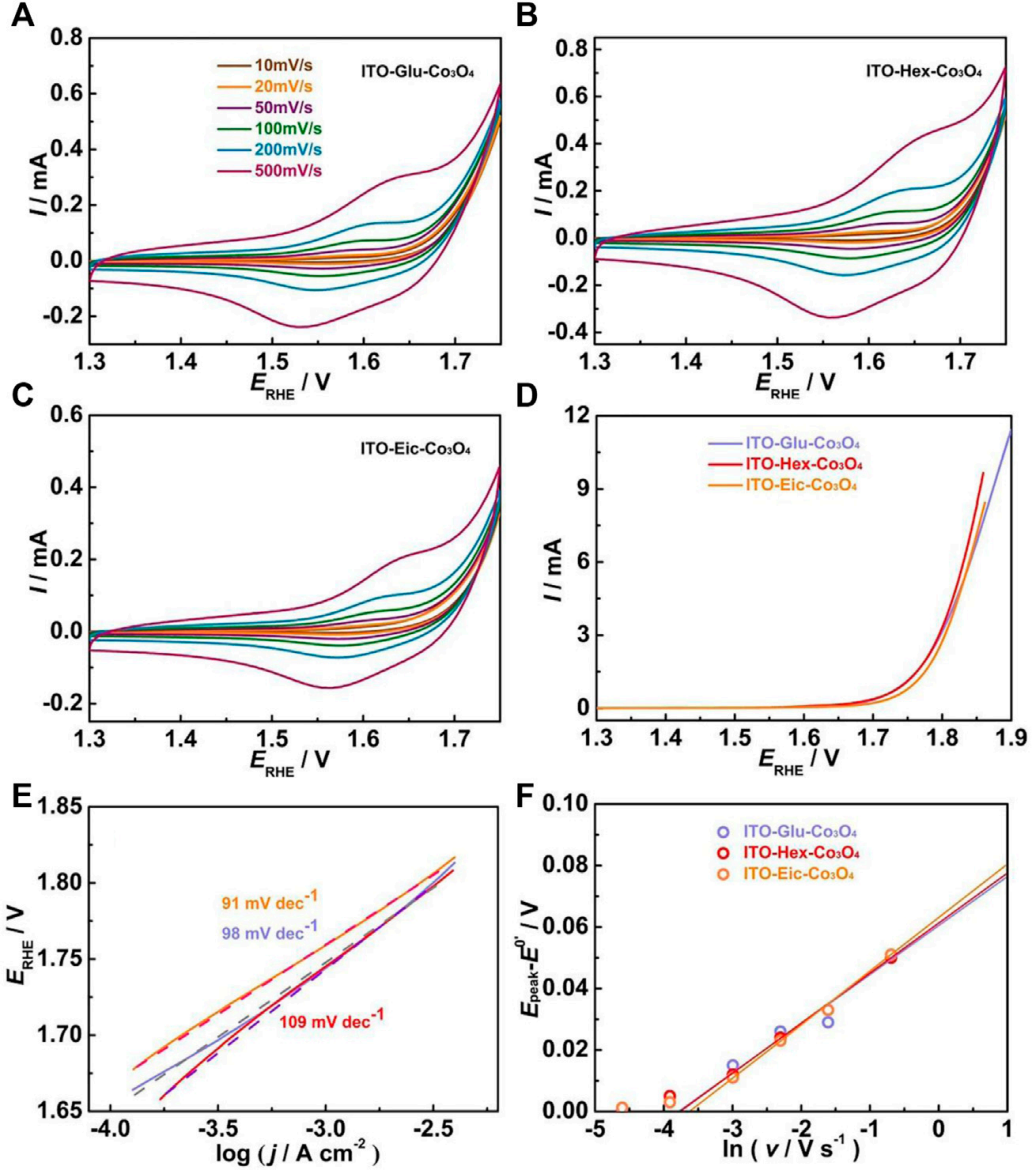
**(A–C)** CV of the ITO-Glu-Co_3_O_4_, ITO-Hex-Co_3_O_4_, and ITO-Eic-Co_3_O_4_ at various scan rates in 0.1 M HClO_4_; **(D)** LSV of these electrodes at 5 mV s^−1^ in 0.1 M HClO_4_; **(E)** Corresponding Tafel plots and the Tafel slope values of these electrodes; **(F)** Plots of anodic (*E*_peak_−*E* ^0’^) against ln *v* of these electrodes.

The surface modification process is further conducted using carboxylic acids with different chain lengths. Figure 5 A–C shows the CV at various scan rates of the ITO-Glu-Co_3_O_4_, ITO-Hex-Co_3_O_4_, and ITO-Eic-Co_3_O_4_, respectively, in 0.1 M HClO_4_. All electrodes show distinct Co^3+/4+^ redox peaks, which suggests the successful attachment of the Co_3_O_4_ to ITO. The CV at 50 mV s^−1^ was used to estimate the *Γ*, and the results are summarized in Table 1. Figure 5D displays the LSV for the ITO-Glu-Co_3_O_4_, ITO-Hex-Co_3_O_4_, and ITO-Eic-Co_3_O_4_ from 1.3–1.9 V at 10 mV s^−1^. All electrodes display electrocatalytic OER activities that originate from the Co_3_O_4_. The corresponding Tafel plots are shown in Figure 5E, and similar Tafel slope values for all three electrodes are observed, which suggests similar OER mechanistic pathways. The Tafel slope values, overpotentials at 1 and 5 mA cm^−2^, and the TOF at 1.72 V of these electrodes are summarized in Table 1. The electron transfer rate constants (*k*) are analyzed using the peak separations from CV at various scan rates based on Laviron’s approach (Lavagnini et al., 2004; Lachmanová et al., 2021). The *E*_peak_−*E* ^0’^ is related to ln *v* by Eq. 2,

$$E_{\text{peak}}-E^{0^{\prime }}=-\frac{RT}{\alpha nF}\text{ln}\left(\frac{\alpha nFv}{RTk}\right),$$ (2)

where *E*_peak_ is the redox peak potential, *E* ^0’^ is the formal redox potential, *α* is the transfer coefficient, *n* is the number of electrons transferred, and other symbols have their standard meanings. By plotting the *E*_peak_−*E* ^0’^ against the ln *v*, the fitted line intercepts with the *x*-axis, and the *k* can be calculated based on Eq. 3.

$$k=\frac{\alpha nFv}{RT}.$$ (3)

Figure 5F shows the *E*_peak_−*E* ^0’^ versus ln *v* plot for the ITO-Glu-Co_3_O_4_, ITO-Hex-Co_3_O_4_, and ITO-Eic-Co_3_O_4_. The calculated *k* values are close and are summarized in Table 1.

**FIGURE 6:**
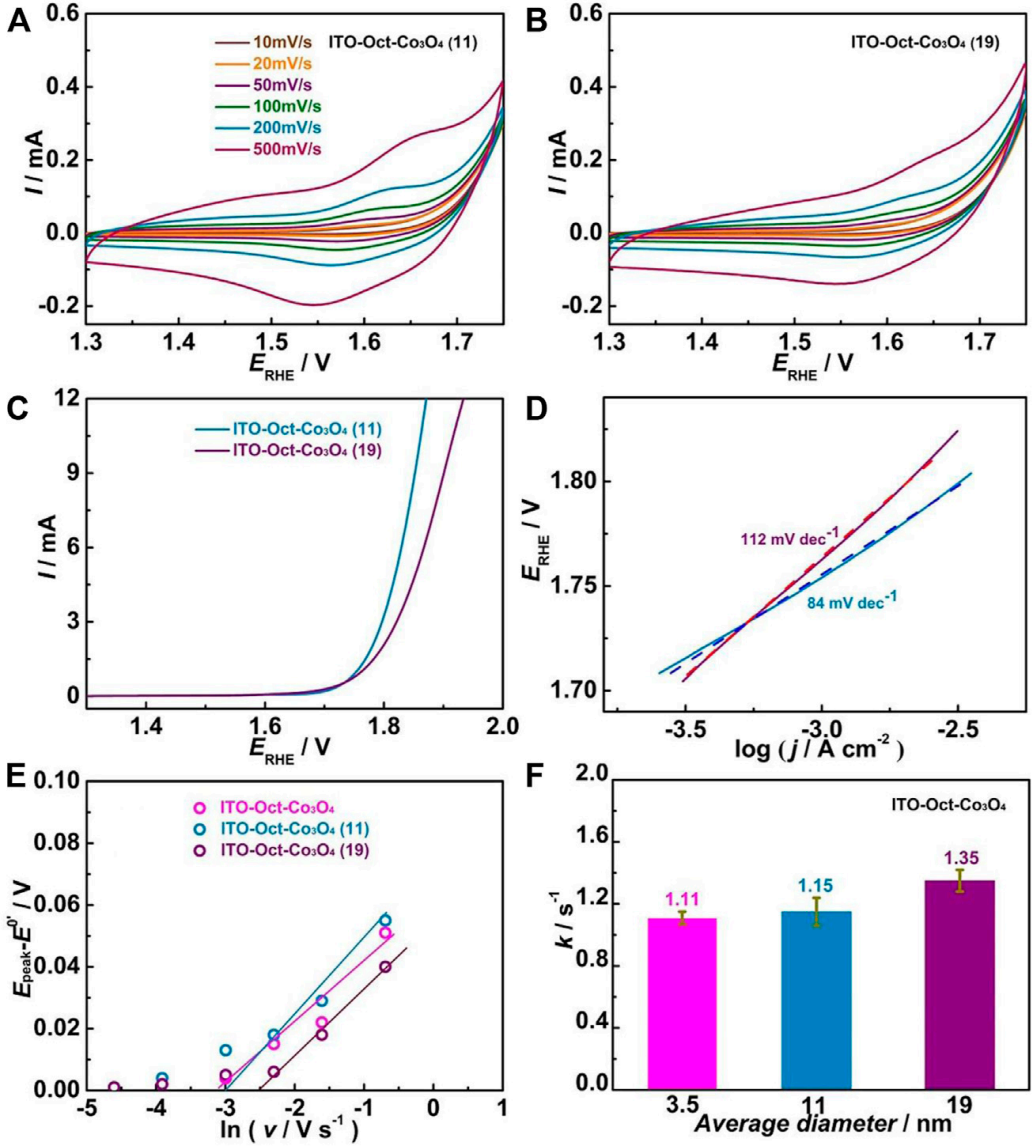
**(A,B)** CV of the ITO-Oct-Co_3_O_4_ (11) and (19) at various scan rates in 0.1 M HClO_4_; **(C)** LSV of these electrodes at 5 mV s^−1^ in 0.1 M HClO_4_; **(D)** Corresponding Tafel plots of these electrodes; **(E)** Plots of anodic (*E*_peak_−*E* ^0’^) against ln *v* of these electrodes; **(F)** *k* versus the average diameter of Co_3_O_4_ of the ITO-Oct-Co_3_O_4_.

By utilizing Co_3_O_4_ with different average diameters, ITO-Oct-Co_3_O_4_ (11) and (19) are constructed. Figures 6A,B display the CV at various scan rates in 0.1 M HClO_4_ for the ITO-Oct-Co_3_O_4_ (11) and (19), respectively. Both electrodes show Co^3+/4+^ redox peaks, and the *Γ* are calculated and summarized in Table 1. Figure 6C shows the LSV of these electrodes. Both electrodes are active toward OER, and the OER onset overpotential and overpotentials to reach 1 and 5 mA cm^−2^ and the TOF at 1.72 V are all summarized in Table 1. Figure 6D shows the Tafel slope of these two electrodes. The Tafel slope values for the ITO-Oct-Co_3_O_4_ (11) and (19) are 112 and 84 mV dec^−1^, respectively. Figure 6E shows the *E*_peak_−*E* ^0’^ versus ln *v* plots for the ITO-Oct-Co_3_O_4_ (11) and (19), together with the ITO-Oct-Co_3_O_4_ (the one with 3.5 nm average diameter. The calculated *k* values are summarized in Figure 6F and in Table 1.

From Table 1, we observe a dependence of *k* on the nanoparticle size, as *k* increases with the increased average diameter of the Co_3_O_4_. Based on the Marcus theory of electron transfer, the *k* is related to the reorganization energy (*λ*) and the extent of electron coupling. For an NP with a radius *r*, the *λ* is related to nanoparticle radius (*r*) by Eq. 4 (Chazalviel and Allongue, 2011),

$$\lambda =\frac{q^{2}}{4\pi \varepsilon_{0}r},$$ (4)

where *q* is the unit charge and *ε*_0_ is the vacuum permittivity. A larger *r* of the nanoparticle would entail a lower *λ*, which increases the rate of electron transfer. The dependence of *k* on the carboxylic acid chain lengths is not obvious as all *k* of the ITO-Glu-Co_3_O_4_, ITO-Oct-Co_3_O_4_, ITO-Hex-Co_3_O_4_, and ITO-Eic-Co_3_O_4_ are in the range of 1.10–1.14 s^−1^. This phenomenon is consistent with Chazalviel’s theory describing the relationship between the insulating layer thickness and the *k* in the substrate–SAM–nanoparticle assembly (Chazalviel and Allongue, 2011). Based on their theory, nanoparticles with 3.5 nm in diameter in the substrate–SAM–nanoparticle assembly would require approximately 20 −CH_2_- units in the carbon chain to reach the critical point, above which electron transfer would become hindered. In our case, no hindered electron transfer is observed using carboxylic acids with 18 or fewer −CH_2_- units. Also, this observation is in accordance with Bard’s model where the tunneling current, though it decreases with increased distance between the substrate surface and the nanoparticle, is still higher than the kinetic current of the Co^3+/4+^ oxidation process. The *k* values reported are close to the literature value where Wang et al. fabricated the electrodeposited Co_3_O_4_ nanosheet on Ti foil in 1 M KOH and observed the *k* at 0.29 s^−1^ (Xiao et al., 2020).

The electrocatalytic OER mechanism of Co_3_O_4_ is proposed through four consecutive proton-coupled electron transfer (PCET) processes in acid as shown in Eqs 5–8 (Shinagawa et al., 2015; Srinivasa et al., 2020; Xiao et al., 2020).

$$\text{Co}+{\text{H}}_{2}\text{O}\to \text{Co}-\text{OH}+{\text{H}}^{+}+{\text{e}}^{-},$$ (5)

$$\text{Co}-\text{OH}\to \text{Co}-\text{O}+{\text{H}}^{+}+{\text{e}}^{-},$$ (6)

$$\text{Co}-\text{O}+{\text{H}}_{2}\text{O}\to \text{Co}-\text{OOH}+{\text{H}}^{+}+{\text{e}}^{-},$$ (7)

$$\text{Co}-\text{OOH}\to \text{Co}+{\text{O}}_{2}+{\text{H}}^{+}+{\text{e}}^{-}.$$ (8)

where Co represents an electrochemically active Co site and the −O, −OH, and −OOH represent the surfaced-adsorbed oxo, hydroxyl, and peroxyl intermediates, respectively. The Tafel slope at 120 mV dec^−1^ represents that the adsorption of -OH is rate-limiting (Eq. 5), while the Tafel slope of 40 mV dec^−1^ suggests that the deprotonation of −OH to form −O is rate-limiting (Eq. 6). Tafel slopes between these two values arise owing to different relative rates of the first two elementary steps in the mechanism. On the Co_3_O_4_ surface, the Tafel slope of our electrodes and literature reported values all lie in the range of 79–120 mV dec^−1^, which suggests that the adsorption of −OH is rate-limiting (Mondschein et al., 2017; Han et al., 2021; Natarajan et al., 2021). This is also consistent with the volcano plots calculated by DFT (Song et al., 2020). Other interpretations of the Tafel slope include the influence of the dissolution of Co_3_O_4_ during the OER that leads to a Tafel slope value that deviates from the typical one (Mondschein et al., 2017).

The TOF of the ITO-SAM-Co_3_O_4_ depends on the carbon chain length of the dicarboxylic acid, with the ITO-Glu-Co_3_O_4_ and the ITO-Oct-Co_3_O_4_ being higher than the ITO-Hex-Co_3_O_4_ and the ITO-Eic-Co_3_O_4_. This might be caused by the decrease of the tunneling current with increased chain lengths of the SAM, which entails the shift from the OER kinetics-controlled current to the mixed tunneling and OER kinetics-controlled current according to Bard’s model. Also, a decrease in the TOF is observed with larger Co_3_O_4_ size. This suggests that the OER activity per active Co site is lower in the case of larger nanoparticles, which is related to the structure of Co_3_O_4_, like crystallinity and surface defects. The OER activity of the constructed ITO-Oct-Co_3_O_4_ is compared to that of other reported Co_3_O_4_ electrocatalysts in acid, and the results are summarized in Supplementary Table S3. However, the stability of the constructed ITO-Oct-Co_3_O_4_ is limited toward OER in acid, which is mainly attributed to the dissolution of active Co sites from Co_3_O_4_ as evidenced by the loss of Co features in CV during repetitive potential cycling (Supplementary Figure S6). ICP-AES on the electrolyte after 1,000 cycles of the potential cycling shows that 13% of Co was leached into the electrolyte. Also, the ITO-Oct-Co_3_O_4_ can only sustain the 0.05 mA cm^−2^ galvanostatic measurement for 2500 s in 0.1 M HClO_4_ (Supplementary Figure S8). In acid, the gradual formation of a porous hydrous oxide layer with a loosely bonded Co center is observed and is proposed to be related to OER stability (Natarajan et al., 2021). The formed hydrous oxide layers could also affect the stability of the anchoring ester groups, which could lead to loss of the Co_3_O_4_. Further optimization of the Co_3_O_4_ structure, like the incorporation of acid stable components (Huynh et al., 2017), is required to enhance the stability of the assembly.

## Conclusion

We constructed the Co_3_O_4_-modified ITO electrodes using dicarboxylic acid as the bridging molecule. The ITO-SAM-Co_3_O_4_ were characterized using electrochemistry and XPS and are active toward the OER in acid. The ITO-Oct-Co_3_O_4_, with Co loading at 3.31 × 10^−8^ mol cm^−2^ exhibits 374 mV onset overpotential and 497 mV overpotential to reach 1 mA cm^−2^ OER current density in 0.1 M HClO_4_. The *k* is not affected by the carbon chain lengths of the SAM, and an increase in the Co_3_O_4_ size enhances the *k*, which is consistent with the previous theory of the electron transfer kinetics. Enhanced TOF of the OER is observed on electrodes with shorter carbon chains and smaller Co_3_O_4_. Meanwhile, the stability of the ITO-SAM-Co_3_O_4_ is limited by the Co_3_O_4,_ which is prone to dissolute under the OER in acid. Strategies for enhancing the stability of the Co_3_O_4_ in acid are essential in developing non-noble metal-based interfaces for OER. In addition, the Co_3_O_4_ tethered to ITO by SAM exhibits significantly higher *Γ* and higher TOF of the OER than the Co_3_O_4_ bound to ITO using Nafion, and we propose that evaluation of the nanoparticle electrocatalytic activities using Nafion as the binder in the electrode preparation would cause severe underestimation. Nevertheless, binding nanoparticle electrocatalysts with Nafion or other ionomers is currently the most practical way of fabricating the membrane electrode assembly in fuel cells and water electrolyzers. The nanoparticle-tethered electrodes are promising as platforms for evaluation of the electrochemical catalytic activities of the nanoparticles.

## Data Availability

The original contributions presented in the study are included in the article/Supplementary Material; further inquiries can be directed to the corresponding authors.
