# Supplementary material for: Electron transfer dynamics and electrocatalytic oxygen evolution activities of the Co3O4 nanoparticles attached to indium tin oxide by self-assembled monolayers
Source: Front Chem. 2022 Aug 24;10:919192. doi: 10.3389/fchem.2022.919192 (PMC9448888; doi:10.3389/fchem.2022.919192)
Supplement: Supplementary file 1 [file DataSheet1.docx]

Electron Transfer Dynamics and Electrocatalytic Oxygen Evolution Activities of the Co_3_O_4_ Nanoparticles Attached to Indium Tin Oxide by Self-Assembled Monolayers

Xuan Liu, Qianhong Tian, Yvpei Li, Zixiang Zhou, Jinlian Wang, Shuling Liu*, Chao Wang*

*Department of Chemistry and Chemical Engineering, Shaanxi Collaborative Innovation Center of Industrial Auxiliary Chemistry & Technology, Key Laboratory of Auxiliary Chemistry and Technology for Chemical Industry, Ministry of Education, Shaanxi University of Science and Technology, Xi’an, Shaanxi 710021, China*

^*^Corresponding author.

*E-mail address*: liushuling@sust.edu.cn

cwang@sust.edu.cn

1. Experimental
2. X-ray diffraction
3. Transmission electron micrographs
4. Electrochemistry
5. X-ray photoelectron spectra
6. Tables

**1. Experimental**

*1.1 Chemicals*

The following chemicals are used in the experiments, and all the reagents were analytically pure: glutaric acid (C_5_H_8_O_4_, Zhengzhou Paini Chemical Reagent Factory), octanedioic acid (C_8_H_14_O_4_, Sinopharm Chemical Reagent Co., Ltd), hexadecanedioic acid (C_16_H_30_O_4_, Shanghai Yuanye Bio-Technology Co., Ltd), eicosanedioic acid (C_20_H_38_O_4_, Zhengzhou Anmusi Chemical Products Co., Ltd), cobalt acetate (Co(CH_3_COO)_2_·4H_2_O, Tianjin Hongyan Reagent Factory), cobalt chloride (CoCl_2_·6H_2_O, Tianjin Hongyan Reagent Factory), acetone (C_3_H_6_O, Rionlon Chemical Reagent), ammonia solution (NH_3_, Guangdong Guanghua Sci-Tech Co., Ltd), ethanol (C_2_H_5_OH, Rionlon Chemical Reagent), Nafion® (5 wt%, Dupont), perchloric acid (HClO_4_, Tianjin Damao) hydrochloric acid (HCl, Sinopharm Chemical Reagent Co., Ltd). Doubly distilled water was used in the experiments.

*1.2 Instrumentation*

X-ray photoelectron spectroscopy (XPS) was carried out using a Kratos Axis Supra spectrometer at room temperature and ultra-high vacuum (UHV) conditions. The instrument was equipped with monochromatic Al Kα source 1486.6 eV (15 mA, 15 kV), and hemispherical analyser with hybrid magnetic and electrostatic lens for enhanced electron collection. Survey and detailed XPS spectra were acquired at normal emission with the fixed pass energy of 160 eV and 40 eV, respectively. Slot aperture 700 × 300 μm^2^ was used during the acquisition. All spectra were charge-corrected to the hydrocarbon peak set to 284.6 eV. The Kratos charge neutralizer system was used on all specimens. Data analysis was based on a standard deconvolution method using mixed Gaussian (G) and Lorentzian (L) line shape (G = 70% and L = 30%, Gaussian–Lorentzian product) for each component. Spectra were analyzed using CasaXPS software (version 2.3.16). X-ray diffraction was acquired using D8 ADVANCE (Bruker) diffractometer having Cu Kα (λ=1.54 Å) source. The instrument was operated at 30 mA current voltage and 40 kV. Bare ITO and ITO-Oct-Co_3_O_4_ are laid flat at the sample holder to acquire the XRD patterns.

The Co loading on the electrode is measured using Thermo Scientific iCAP 6300 Inductively Coupled Plasma-Atomic Emission Spectrometers (ICP-AES). The ITO-Oct-Co_3_O_4_ is first immersed in concentrated nitric acid for 2 h, and solution is then diluted and neutralized using KOH. The Co introduced by the nitic acid and KOH are subtracted. ICP-AES parameters are the following: forward power 1350 W, plasma gas flow rate 12.0 L min^−1^, nebulizer gas flow rate 1.0 L min^−1^, auxiliary gas flow rate 1.0 L min^−1^, sample uptake speed 50 rpm with white/orange Tygon tubing. A concentric nebulizer was used with a cyclonic spray chamber. No internal standard correction was applied for ICP-AES analysis. Transmission electron microscope (TEM) was used FEI Talos F200X.

**2. X-ray Diffraction**

**
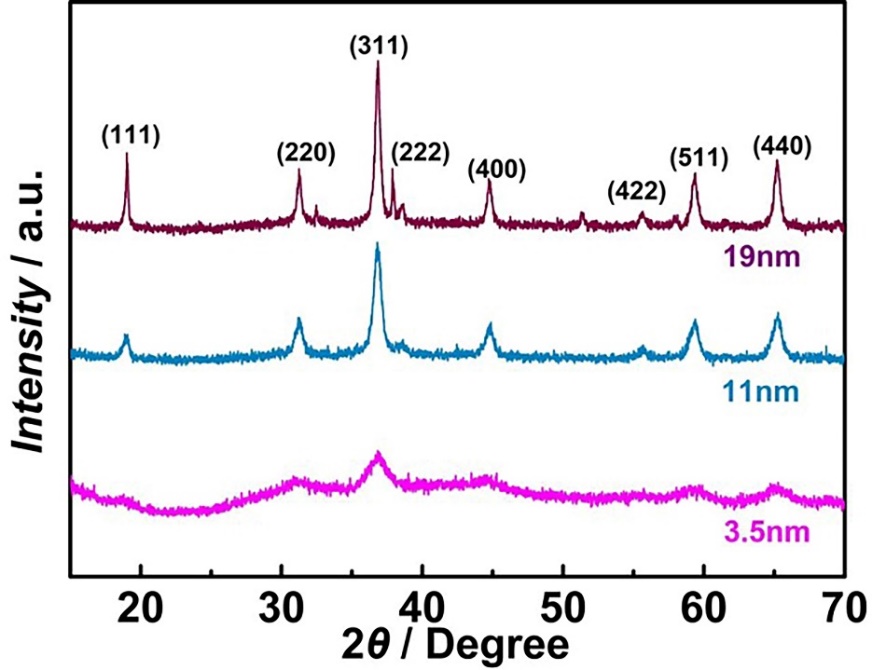
**

**Fig. S1.** XRD patterns of Co_3_O_4_ nanoparticles with various sizes.

**
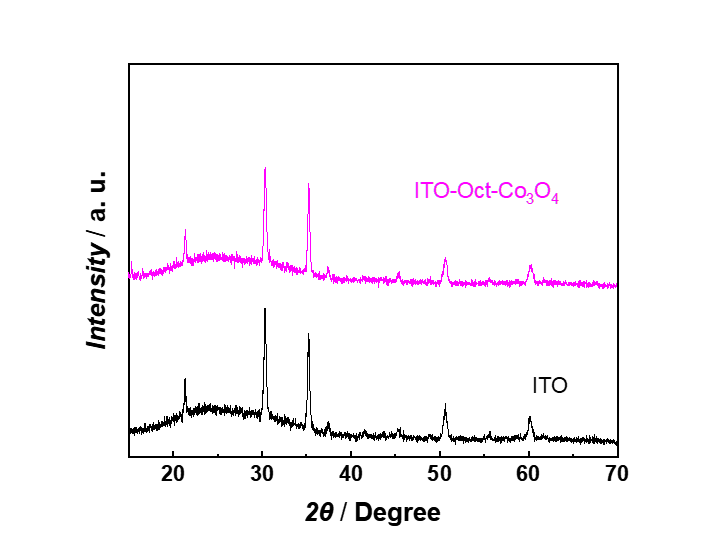
**

**Fig. S2.** XRD patterns of the ITO-Oct-Co_3_O_4_ and the bare ITO.

**3.** **Transmission electron micrographs**


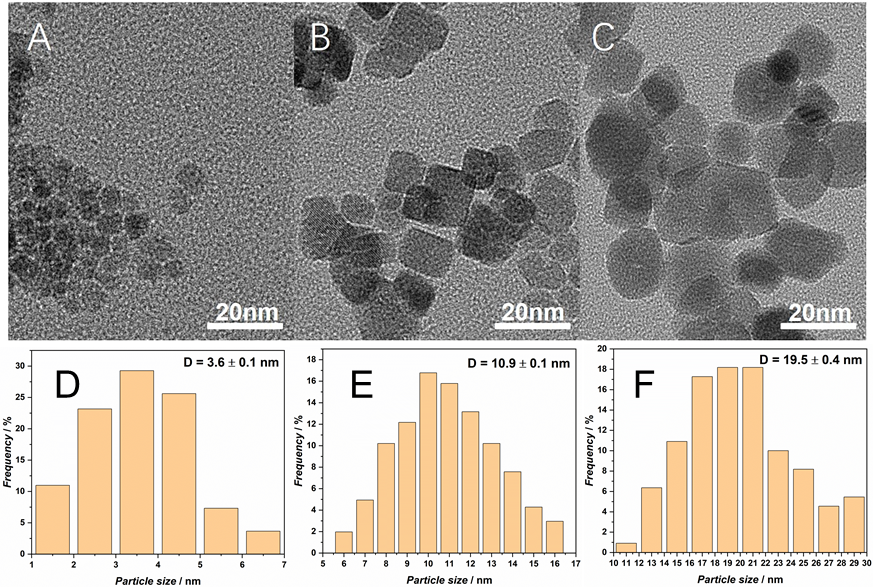


**Fig. S3.** TEM image of the Co_3_O_4_ nanoparticles (A) 3.5 nm, (B) 11 nm, and (C) 19 nm.

**4. Electrochemistry**


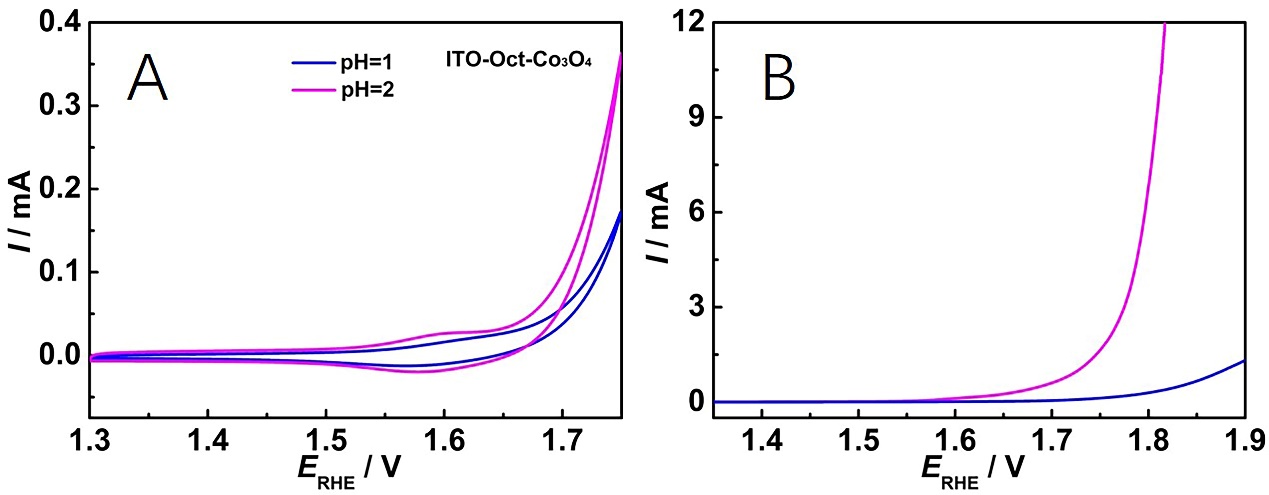


**Fig. S4.** (A) CV (50 mV s^-1^) and (B) LSV (5 mV s^-1^) of the ITO-Oct-Co_3_O_4_ in 0.1 M HClO_4_. The pH = 1 or 2 is the pH of the Co_3_O_4_ colloidal solutions during the metalation process to fabricate the ITO-Oct-Co_3_O_4_.

The effect of the pH of the Co_3_O_4_ colloidal solution on the electrochemical properties of the ITO-Oct-Co_3_O_4_ is investigated. Fig. S4 show the CV and LSV in 0.1 M HClO_4_ of the ITO-Oct-Co_3_O_4_ prepared using the pH = 1 or 2 Co_3_O_4_ colloidal solutions. The electrode prepared with pH = 2 Co_3_O_4_ colloidal solution exhibits higher Co^3+/4+^ redox peaks and higher OER current densities. This suggests that with pH = 2 solution, higher Co_3_O_4_ coverage on ITO can be achieved through the esterification reaction between the hydroxyl groups on the Co_3_O_4_ surface with the carboxylic acid group. Though the [H^+^] can catalyze the surface esterification reaction, high concentration of [H^+^] could also lead to hydrolysis of the surface ester bond, and lead to decreased surface coverage of Co_3_O_4_ at pH = 1. Also, the lateral electrostatic repulsion between the Co_3_O_4_ nanoparticles is also affected by the pH of the solution. Highly acidic solution may lead to enhanced positive surface charges at the Co_3_O_4_ nanoparticles, and to greater repulsion and lower surface coverage. Similar phenomenon is also observed for ITO-citric acid-IrO_x_. Taking both factors into consideration, we determine that the pH = 2 of the Co_3_O_4_ colloidal solution yields the highest coverage of the Co_3_O_4_ on the electrode.


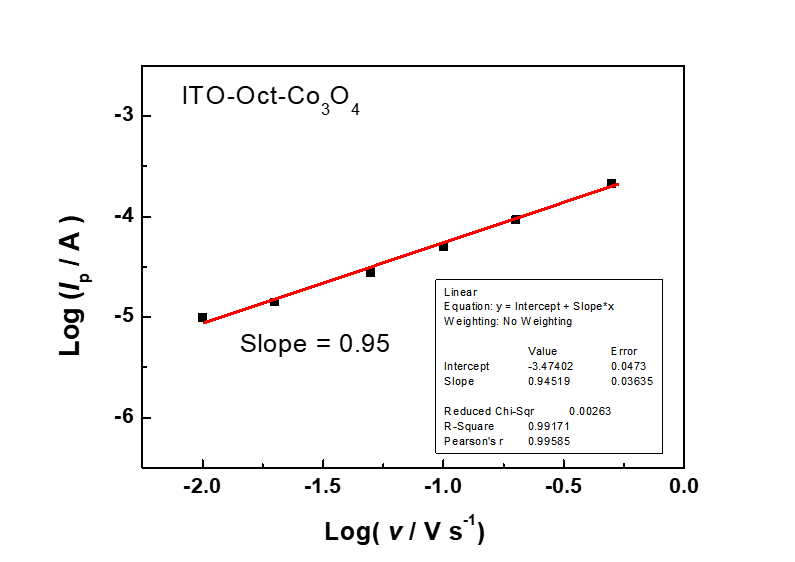


**Fig. S5.** The fitted plot of log *i*_p_ (oxidation peak current) versus log *v* of the ITO-Oct-Co_3_O_4_.


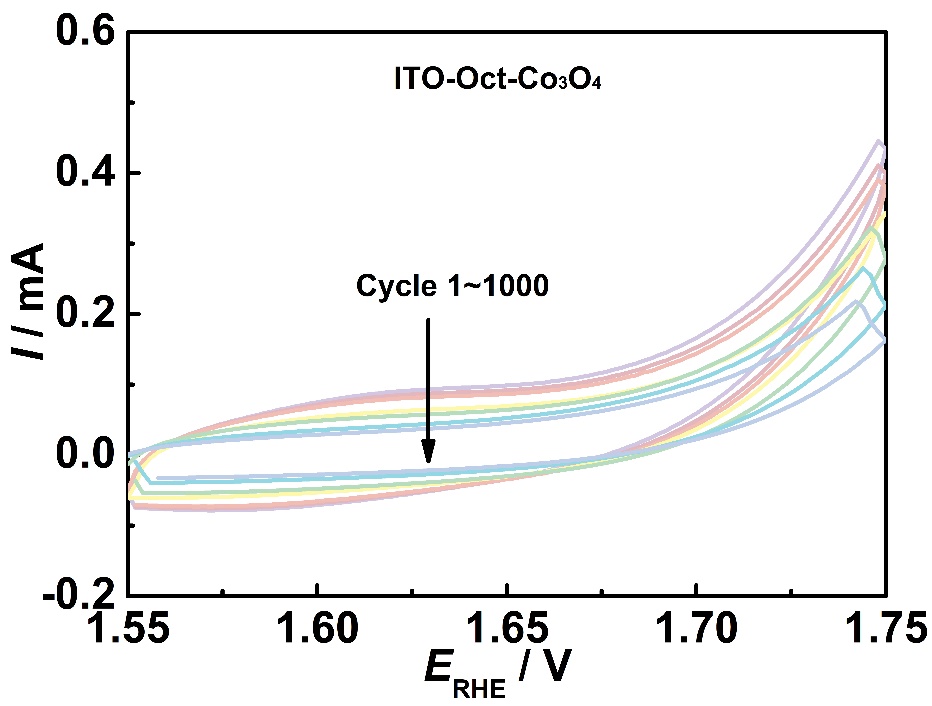


**Fig. S6.** CV for the ITO-Oct-Co_3_O_4_ cycled between 1.55 V and 1.75 V at 200 mV s^-1^ in 0.1 M HClO_4_.


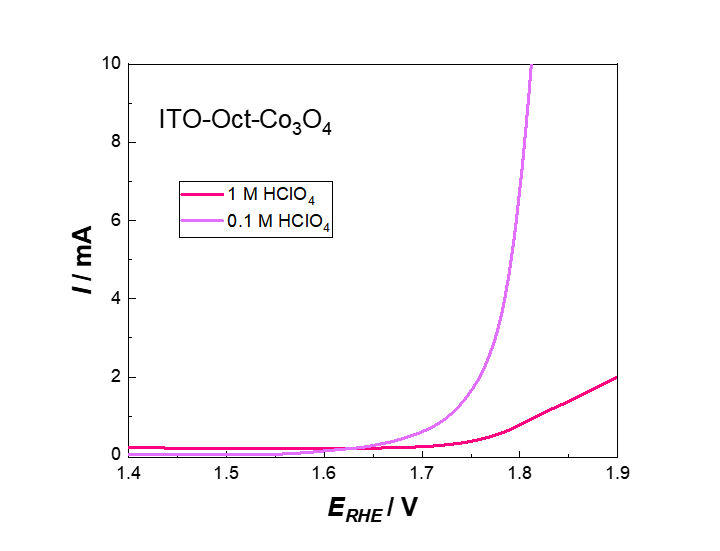


**Fig. S7.** LSV of the ITO-Oct-Co_3_O_4_ at 5 mV s^-1^ in 0.1 M HClO_4_ and 1 M HClO_4_.


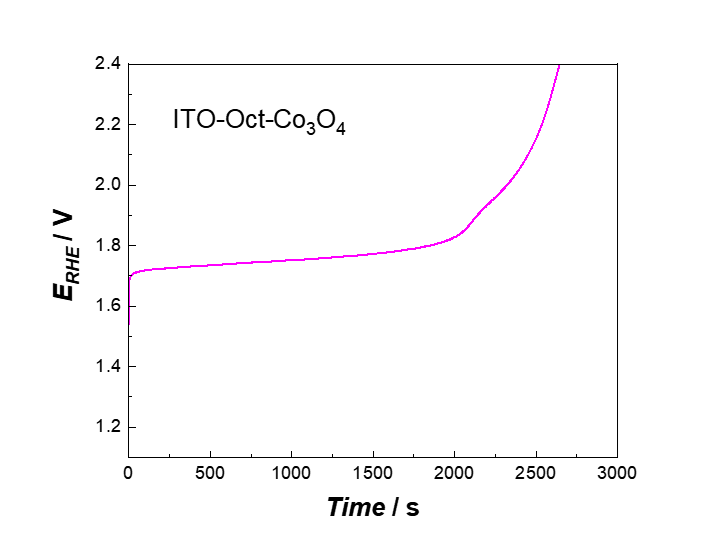


**Fig. S8.** Galvanostatic result at 0.05 mA cm^-2^ of the ITO-Oct-Co_3_O_4_ in 0.1 M HClO_4_.

**5. X-ray photoelectron spectra**


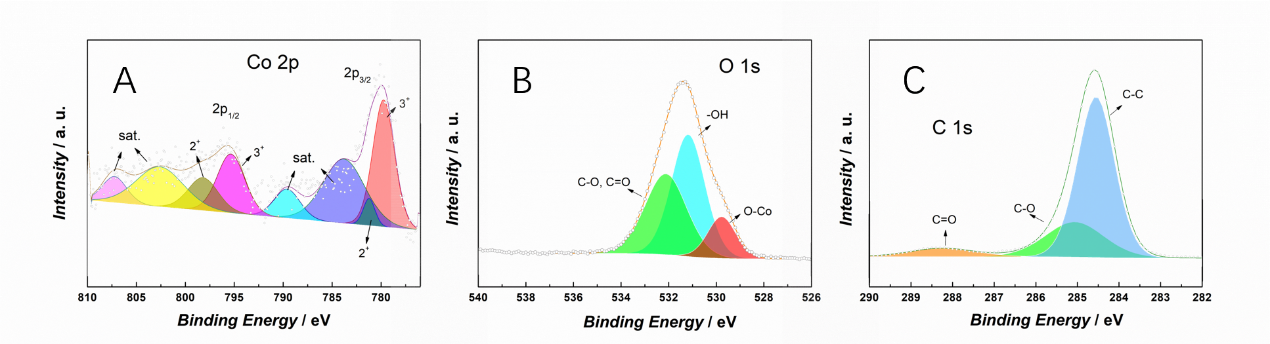


**Fig. S9.** Deconvoluted XPS spectra of the ITO-Oct-Co_3_O_4_ after 1000 cycles in 0.1 M HClO_4_.

**6. Tables**

**Table S1.** Amounts of the reactants to synthesize Co_3_O_4_ nanoparticles.

|  | Co(ac)_2_·4 H_2_O / g | C_2_H_5_OH / mL | H_2_O / mL | NH_3_·H_2_O / mL |
| --- | --- | --- | --- | --- |
| 3.5 nm Co_3_O_4_ | 0.5 | 25 | 0 | 2.5 |
| 11 nm Co_3_O_4_ | 0.5 | 15 | 10 | 2.5 |
| 19 nm Co_3_O_4_ | 0.5 | 0 | 25 | 2.5 |

**Table S2.** The concentration of the acid baths.

|  | ITO-Glu-Co_3_O_4_ | ITO-Oct-Co_3_O_4_ | ITO-Hex-Co_3_O_4_ | ITO-Eic-Co_3_O_4_ |
| --- | --- | --- | --- | --- |
| *c*(acid)  /mol L^-1^ | 0.076 | 0.126 | 0.056 | 0.029 |

**Table S3.** Electrochemical performance of Co-based OER catalysts under similar conditions reported in recent literatures.

| Samples | Electrolyte | Co loading / mg cm^-2^ | *η*_onset_ /mV | *η*@10mA cm^-2^/mV | Ref. |
| --- | --- | --- | --- | --- | --- |
| ITO-Oct-Co_3_O_4_ | 0.1 M HClO_4_ | 0.002 | 374 | 570 | This work |
| Co_3_O_4_/FTO | 0.5 M H_2_SO_4_ | 0.134 | 393 | 570 | [1] |
| Co_2_TiO_4_/CC | 0.5 M H_2_SO_4_ | 2.692 | 69 | 513 | [2] |
| Co_3_O_4_/CC | 0.5 M H_2_SO_4_ | 0.013 | 210 | 839 | [2] |
| Co_3_O_4_ @C/CP | 0.5 M H_2_SO_4_ |  | 320 | 370 | [3] |
| Co_0.05_Fe_0.95_O_y_ | 0.5 M H_2_SO_4_ |  | 440 | 650 | [4] |
| Ba[Co-POMs] | 1 M H_2_SO_4_ | 11 | 88 | 361 | [5] |
| Co_3_O_4_ | 1 M H_2_SO_4_ | 19 | 90 | 410 | [5] |
| HNC-Co | 0.5 M H_2_SO_4_ | 0.001 | 100 | 265 | [6] |
| Co/29BC | 0.5 M H_2_SO_4_ |  | 190 | 450 | [7] |

**References**

[1] J.S. Mondschein, J.F. Callejas, C.G. Read, J.Y.C. Chen, C.F. Holder, C.K. Badding, R.E. Schaak, Crystalline cobalt oxide films for sustained electrocatalytic oxygen evolution under strongly acidic conditions, Chemistry of Materials, 29 (2017) 950-957.

[2] S. Anantharaj, K. Karthick, S. Kundu, Spinel cobalt titanium binary oxide as an all-non-precious water oxidation electrocatalyst in acid, Inorganic Chemistry, 58 (2019) 8570-8576.

[3] X. Yang, H. Li, A.-Y. Lu, S. Min, Z. Idriss, M.N. Hedhili, K.-W. Huang, H. Idriss, L.-J. Li, Highly acid-durable carbon coated Co_3_O_4_ nanoarrays as efficient oxygen evolution electrocatalysts, Nano Energy, 25 (2016) 42-50.

[4] W.L. Kwong, C.C. Lee, A. Shchukarev, J. Messinger, Cobalt-doped hematite thin films for electrocatalytic water oxidation in highly acidic media, Chemical Communications, 55 (2019) 5017-5020.

[5] M. Blasco-Ahicart, J. Soriano-López, J.J. Carbó, J.M. Poblet, J.-R. Galan-Mascaros, Polyoxometalate electrocatalysts based on earth-abundant metals for efficient water oxidation in acidic media, Nature chemistry, 10 (2018) 24-30.

[6] H. Su, X. Zhao, W. Cheng, H. Zhang, Y. Li, W. Zhou, M. Liu, Q. Liu, Hetero-N-coordinated Co single sites with high turnover frequency for efficient electrocatalytic oxygen evolution in an acidic medium, ACS Energy Letters, 4 (2019) 1816-1822.

[7] Q. Lai, V. Vediyappan, K.-F. Aguey-Zinsou, H. Matsumoto, One‐step synthesis of carbon‐protected Co_3_O_4_ nanoparticles toward long‐term water oxidation in acidic media, Advanced Energy and Sustainability Research, 2 (2021) 2100086.
